# Non-capsulated and capsulated *Haemophilus influenzae *in children with acute otitis media in Venezuela: a prospective epidemiological study

**DOI:** 10.1186/1471-2334-12-40

**Published:** 2012-02-15

**Authors:** Laura Naranjo, Jose Antonio Suarez, Rodrigo DeAntonio, Francis Sanchez, Alberto Calvo, Enza Spadola, Nicolás Rodríguez, Omaira Andrade, Francisca Bertuglia, Nelly Márquez, Maria Mercedes Castrejon, Eduardo Ortega-Barria, Romulo E Colindres

**Affiliations:** 1 Policlínica Metropolitana, Piso 1, consultorio 1-13, Urbanización Caurimare, Calle A-1, Caracas, Venezuela; 2 GlaxoSmithKline Biologicals, Clayton, Ciudad del Saber Edificio 230, Panama City, Panama; 3 Laboratorio Metropolitano, Sotano 2, Urbanización Caurimare, Calle A-1, Caracas, Venezuela; 4 Instituto Nacional de Higiene, Ciudad Universitaria Los Chaguaranos, Caracas, Venezuela; 5 GlaxoSmithKline Biologicals, Estrada dos Bandeirantes, 8.464 Jacarepaguá 22783-110 Rio de Janeiro, Brazil; 6 GlaxoSmithKline Biologicals, Avenue Fleming 20, B-1300 Wavre, Belgium

**Keywords:** Acute otitis media, Non-typeable *Haemophilus influenzae *(NTHi), Pneumococcal conjugate vaccine

## Abstract

**Background:**

Non-typeable *Haemophilus influenzae *(NTHi) and *Streptococcus pneumoniae *are major causes of bacterial acute otitis media (AOM). Data regarding AOM are limited in Latin America. This is the first active surveillance in a private setting in Venezuela to characterize the bacterial etiology of AOM in children < 5 years of age.

**Methods:**

Between December 2008 and December 2009, 91 AOM episodes (including sporadic, recurrent and treatment failures) were studied in 87 children enrolled into a medical center in Caracas, Venezuela. Middle ear fluid samples were collected either by tympanocentesis or spontaneous otorrhea swab sampling method. Standard laboratory and microbiological techniques were used to identify bacteria and test for antimicrobial resistance. The results were interpreted according to Clinical Laboratory Standards Institute (CLSI) 2009 for non-meningitis isolates. All statistical analyses were performed using SAS 9.1 and Microsoft Excel (for graphical purposes).

**Results:**

Overall, bacteria were cultured from 69.2% (63 of the 91 episodes); at least one pathogen (*S. pneumoniae, H. influenzae, S. pyogenes *or *M. catarrhalis*) was cultured from 65.9% (60/91) of episodes. *H. influenzae *(55.5%; 35/63 episodes) and *S. pneumoniae *(34.9%; 22/63 episodes) were the most frequently reported bacteria. Among *H. influenzae *isolates, 62.9% (22/35 episodes) were non-capsulated (NTHi) and 31.4% (11/35 episodes) were capsulated including types d, a, c and f, across all age groups. Low antibiotic resistance for *H. influenzae *was observed to amoxicillin/ampicillin (5.7%; 2/35 samples). NTHi was isolated in four of the six *H. influenzae *positive samples (66.7%) from recurrent episodes.

**Conclusions:**

We found *H. influenzae *and *S. pneumoniae *to be the main pathogens causing AOM in Venezuela. Pneumococcal conjugate vaccines with efficacy against these bacterial pathogens may have the potential to maximize protection against AOM.

## Background

Acute otitis media (AOM) is one of the most frequently diagnosed bacterial infections in children following nasopharyngeal colonization [1]. It results in frequent pediatric visits both in the developed [2] and developing world [3]. Approximately, three in four children develop at least one episode of AOM by 3 years of age, [4] with peak incidences observed among 6-18 month-old children [2]. A report on the epidemiology of AOM by Block and colleagues [5] showed that 64% of children had experienced their first episode by 6 months, and 86% by 1 year of age; 31% had > 3 episodes in < 12 months of age. AOM is, thus, one of the main causes of childhood morbidity in both developed and developing countries [6]. The estimated annual disease burden of AOM ranges between 8,200,000-12,900,000 cases among children < 5 years of age in Latin America and the Caribbean [7]. In Venezuela, approximately 100,000-130,000 AOM cases per year (2004-2007) are reported in children < 5 years of age (Ministerio del Poder Popular para la Salud. Gobierno Bolivariano de Venezuela, Personal communication, 2010).

AOM is a multi-pathogen disease [8]. *Streptococcus pneumoniae *(*S. pneumoniae*) and non-typeable *Haemophilus influenzae *(NTHi) have consistently been reported as the two major bacterial pathogens responsible for AOM, [9-11] identified in up to 80% of cases. Other regularly reported bacterial pathogens include *Moraxella catarrhalis *(*M. catarrhalis*) and *Streptococcus pyogenes *(*S. pyogenes*) [11]. *S. pneumoniae *has been shown to be responsible for 43.8%-61.4% of all bacterial AOM cases in Latin America [12-14]. *H. influenzae *has, however, emerged as the most frequently recovered bacterial pathogen in recent studies [13,15]. AOM caused by NTHi has been particularly associated with older age groups and recurrent disease [11]. Recurrent to chronic AOM may predispose to chronic suppurative otitis media (CSOM) in a third of cases [16]. Globally, 65-330 million people may suffer from CSOM [17].

Recurrent disease in children has important implications for AOM treatment. Although some cases resolve spontaneously, the majority require antibiotic treatment for early recovery. AOM constitutes the primary motive for the prescription of antibiotics in children [18]. The increasing use of these antibiotics may be acting as a selective pressure driving antibiotic resistance [19]. AOM is the primary cause of antibiotic consumption in Latin America and the Caribbean [20]. Antibiotic consumption in Latin America is highest in Argentina and Venezuela [21]. High prevalence of drug-resistant pathogens like penicillin-resistant *S. pneumoniae *and β-lactamase producing or other ampicillin-resistant *H. influenzae *have become major factors in complicating the management of AOM [22]. Efforts to prevent infectious diseases through vaccination may be able to reduce the demand for antibiotics in the community and the selective persistence of nasopharyngeal bacterial pathogens [23].

Pneumoccocal conjugate vaccines (PCVs) have been shown to be safe and effective in preventing invasive pneumococcal disease (IPD) in children [24,25]. The 7-valent pneumococcal vaccine (PCV-7; *Prevnar*™/*Prevenar*™, Pfizer/Wyeth, US) was introduced into the US public immunization strategy in 2000 and has demonstrated noticeable efficacy against IPD [26,27]; 57% (95%CI: 44-67) efficacy against vaccine-type pneumococcal AOM [28] and overall 7% (95%CI: 4.1-9.7) efficacy against clinical AOM episodes [24]. Wide use of PCV-7 led to a relative shift in the proportion of pathogens causing persistent AOM was observed (decrease in *S. pneumoniae *and an increasing predominance of NTHi) [10]. Furthermore, with widespread vaccination, the predominant cause of remaining cases of pneumococcal disease became non-vaccine serotypes of *S. pneumoniae *[29,30], due to changing antimicrobial resistance patterns and secular trends. There are two new PCVs licensed recently in Venezuela. The 10-valent pneumococcal/*H. influenzae *protein D conjugated vaccine (PHiD-CV, *Synflorix*™, GlaxoSmithKline [GSK] Biologicals, Rixensart, Belgium) has 8 of the 10 serotypes conjugated to a recombinant form of protein D of *H. influenzae*. A randomized, double-blind study with an 11-valent prototype vaccine relying on the protein D carrier provides evidence that proteinD-containing vaccines may offer additional protection (35%) against AOM episodes caused by both pneumococcus and *H. influenzae *[31]. A 13-valent pneumococcal vaccine formulation (PCV-13) is also available.

In Venezuela, *Haemophilus influenzae type B *was introduced into the expanded program of immunization in 2000 [32]. Pneumoccocal conjugate vaccines are not indicated for universal vaccination, however since 2004, PCV-7 has been gradually introduced into private practice. Data on AOM in Latin America are limited and no there are no data on AOM etiology in Venezuela. This study thus provides novel data on the bacterial etiology of AOM and the associated serotypes in Venezuelan children. This study assessed the microbiology of AOM cases, antibiotic susceptibility pattern of the middle ear pathogens obtained from these cases, and the proportion of AOM episodes that may be vaccine-preventable with new pneumococcal vaccines recently licensed.

## Methods

### Study design and subjects

We conducted a prospective epidemiological study within a routine, private clinical setting in a medical center in Caracas, Venezuela from December 2008 to December 2009. Children ≥3 months and < 5 years of age, visiting physician/pediatrician clinics for AOM and for whom a MEF sample was available, were enrolled in the study. We included children presenting with one of the general signs of otalgia/irritability, conjunctivitis, fever and either Paradise's criteria (bulging, diffused or localized inflamed tympanic membranes) or spontaneous otorrhea (< 24 h). Exclusion criteria included: children hospitalized at the time of the diagnosis or treatment of AOM, presenting with a new episode of AOM but having received antibiotic treatment in the previous 72 h for a different illness, those who had received antibiotic by the physician/ear, nose and throat (ENT) specialist prior to tympanocentesis and those with otorrhea for > 24 h or with otitis media with effusion (not AOM).

Two specific groups of children were eligible for study recruitment: - Children with a new episode of AOM (< 72 h of onset) who were yet to receive antibiotic therapy for the episode, and - Children with a diagnosis of AOM within 48-72 h before study enrolment, who had received antibiotic therapy, but remained symptomatic at the time of study entry (treatment failures).

A child could be enrolled multiple times in the study whenever he/she reported a new episode. A case was considered as a new episode if there was a 30-day symptom-free interval since the resolution of the first episode. A recurrent AOM episode was the third or greater new episode within the last 6 months or the fourth or greater new episode within the last 12 months.

The primary endpoints were occurrence of *H. influenzae, S. pneumoniae, M. catarrhalis *and *S. pyogenes *isolated from MEF samples. Secondary endpoints were occurrence of *H. influenzae *and *S. pneumoniae *specific serotypes from MEF samples and antimicrobial susceptibility of all bacterial pathogens isolated from MEF samples.

Written informed consent was obtained from the parents/guardians of participating children before conducting any study-related procedures. We collected demographic information including age and sex assuring confidentiality for subjects enrolled.

### Bacterial identification and characterization

Samples of MEF from all children were collected by an ENT specialist by performing tympanocentesis or by careful sampling of spontaneous otorrhoea (removal and cleaning of the ear canal material and deep aspiration of the MEF material via needle insertion).

### Microbiology

MEF samples were inoculated into Amies transport medium with charcoal [33], sent to the study laboratory (Laboratorio Metropolitano) within 16-48 h, inoculated onto chocolate and blood agar and incubated at 37°C in a 5% CO_2 _atmosphere. For identification, standard guidelines described by the American Society of Microbiology were used and confirmed by using NH and GP cards VITEK-2 compact system (bioMérieux™).

### Clinical feature

A detailed medical history including symptoms, seasonality, vaccination status for PCV and HiB, AOM episodes characteristics including recurrency, A clinical examination was performed recording all data in the study database.

### Antibacterial susceptibility

Antimicrobial susceptibility was done using AST-P68 (bioMérieux™) for *S. pneumoniae*, standard methods for *H. influenzae *and *M. catarrhalis *were according to Clinical Laboratory Standards Institute (CLSI-M100-S19) 2009 and for quality control, *S. pneumoniae ATCC *49619 and *H. influenzae ATCC *49247 y 49766 [34] strains were used. Serotyping for *S. pneumoniae *was done at the national reference laboratory, Laboratorio de Microbiologia del Instituto Nacional de Higiene "Rafael Rangel" using Quellung reaction following SIREVA II guidelines [35]. *H. influenzae *isolates were confirmed by polymerase chain reaction (PCR) procedures and β-lactamase testing (nitrocefin disk) was performed.

### Statistical analyses

We performed the analyses only on the ATP cohort (subjects meeting all eligibility criteria, complying with the protocol-defined procedures, with no elimination criteria during the study and for whom laboratory results of the MEF samples was available). The study results were presented as episodes of AOM.

We calculated the proportions of AOM caused by *S. pneumoniae, H. influenzae *and other bacterial pathogens. Distribution analyses of *S. pneumoniae *and *H. influenzae *serotypes by age, procedure and pneumococcal vaccination status according to age (fully vaccinated, partially vaccinated and unvaccinated) for AOM cases were performed. We also determined seasonality of AOM cases and other isolates. Qualitative variables, including symptoms and vaccination, were compared to explore differences among capsulated and non capsulated *H. influenzae *status with the Fisher exact test. All tests were 2 sided, and the level of significance was set at p < 0.05.

All statistical analyses were performed using Statistical Analysis System (SAS) version 9.1 (or higher) and Microsoft Excel (for graphical purposes).

### Ethics

The study was conducted according to Good Clinical Practice guidelines, the Declaration of Helsinki and the local rules and regulations of the country. All the study-related documents were reviewed and approved by the local Independent Ethics Committee/Institutional Review Board and the study adhered to applicable local guidelines.

## Results

### Demographic characteristics and sample description

We collected data on 92 episodes of AOM reported from 87 enrolled children. Ninety-one episodes were retained in the ATP (according-to-protocol) cohort due to one child being enrolled more than once for the same AOM episode. Only one of these episodes was classified as a treatment failure. Of the 87 children included in the ATP cohort, 84 reported a single episode, two reported two episodes and one child reported three episodes. Any consecutive episode occurred with an interval of > 30 days after the previous episode.

The mean age of the children for all episodes was 28.3 months; the proportion of females was 51.6% (47/91 episodes) (Additional file 1: Table S1).

The majority of the episodes (57.2%; 52/91) were reported in the 1-3 years age group; 30.8% (28/91 episodes) and 26.4% (24/91 episodes) occurred in the 12-23 months and 24-35 months age groups, respectively.

We collected one MEF (middle ear fluid) sample from each of the 91 episodes. Most samples (90.1%; 82/91 episodes) were collected by tympanocentesis, while nine samples (9.9%) were collected from spontaneous otorrhea episodes. Of the nine episodes with spontaneous otorrhea, 18.2% (2/11), 17.9% (5/28) and 8.3% (2/24) were distributed in the 3-11 months, 12-23 months and 24-35 months age groups, respectively.

### Bacterial etiology of episodes

We observed pathogenic bacterial growth in 67.1% (55/82 episodes) and 88.9% (8/9 episodes) of samples collected by tympanocentesis and spontaneous otorrhoea, respectively. Overall, bacteria were cultured from 69.2% (63) of the 91 episodes; one treatment failure episode of AOM did not show any bacterial growth. Overall, 65.9% (60) of the episodes yielded positive cultures for at least one of the four pathogenic bacteria. The most commonly isolated bacteria among MEF samples with bacterial growth were *H. influenzae *and *S. pneumoniae*, detected in 55.5% (35/63) and 34.9% (22/63) of episodes, respectively. Only 3.2% (2/63) episodes were positive for *S. pyogenes*, and 1.6% (1/63 episodes) for *M. catarrhalis*. No single episode was positive for both *S. pneumoniae *and *H. influenzae*.

**Figure 1:**
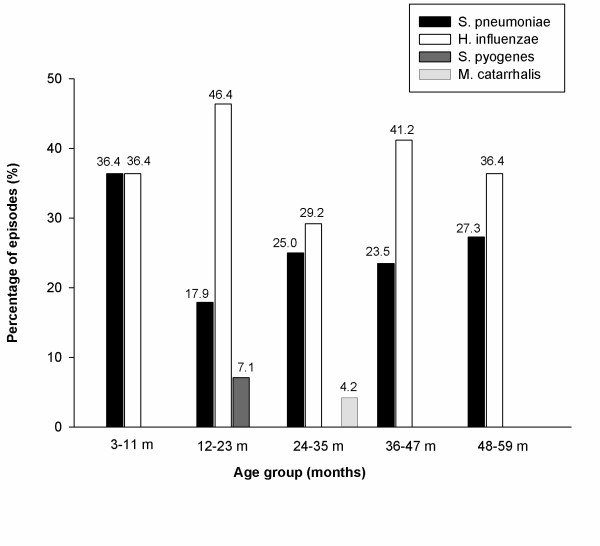
**Bacterial etiology of episodes by age (ATP Cohort)**. The simple bars represent the percentage of bacterial episodes in each age group.

The highest number of positive episodes for at least one of the four pathogenic bacteria under study was in the 3-11 months age group (72.7%; 8/11 episodes); the lowest number of positive episodes was found in the 24-35 months age group (58.3%; 14/24 episodes). The most frequently isolated bacteria across all age groups were *H. influenzae *and *S. pneumoniae *(Figure 1).

Among episodes with spontaneous otorrhea, *S. pneumoniae *was isolated in three episodes, *S. pyogenes *in two, *H. influenzae *in one episode; the remaining two episodes corresponded to other agents.

Recurrent AOM episodes were reported for 5.4% (14/91 episodes) during the study. We obtained bacterial cultures from samples from 71.4% (10/14) of these recurrent episodes. Of these, 6/10 were positive for *H. influenzae*; 3/10 for *S. pneumoniae *and 1/10 was positive for *M. catarrhalis*.

### Bacterial etiology by serotype

Most *H. influenzae *strains were non-capsulated (NTHi: 62.9%; 22/35 episodes). This was followed by serotype *d *(17.1%; 6/35 episodes), *a *and *c *(each accounting for 5.7%; 2/35 episodes), and *f *(2.9%; 1/35 episodes). Two samples (5.7%; 2/35 episodes) were reported as unknown serotype.

**Figure 2:**
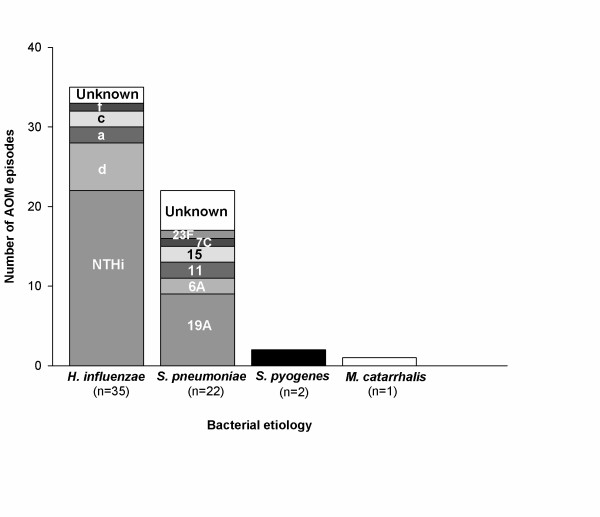
**Bacterial etiology by serotypes (ATP Cohort)**. The stacked column bars represent the number of serotypes for each bacterial positive episode.

Serotype 19A was the most frequently isolated *S. pneumoniae *serotype (40.9%; 9/22 episodes) followed by serotypes 6A, 11 and 15 (each accounting for 9.1%; 2/22 episodes), and 23 F and 7 C (each accounting for 4.5%; 1/22 episodes). Five samples (22.7%; 5/22 episodes) were reported as unknown (Figure 2).

A similar trend of serotype distribution was observed across the age groups for both *H. influenzae *and *S. pneumoniae*.

Of the six *H. influenzae *positive samples from recurrent episodes, NTHi was isolated in four samples (66.7%; 4/6 samples). The remaining two samples corresponded to types c and a. The three *S. pneumoniae *positive samples from recurrent episodes corresponded to serotypes 7 C, 23 F and one was reported as unknown. Of the 14 recurrent AOM episodes, the highest numbers of episodes were reported in the 24-35 months and 36-47 months age groups (5 episodes each); three episodes were reported in the 12-23 months and one episode in the 3-11 months age groups.

### Common symptoms reported

Fever (65.9%; 60/91 episodes) and ear pain (57.1%; 52/91 episodes) were the most common symptoms reported before the study visit and also during the study visit (49.5%; 45/91 episodes and 56%; 51/91 episodes, for fever and ear pain respectively). We did not observe significant differences between capsulated and non-capsulated *H. influenzae *for fever (68.2% vs. 72.7%; *p *= 0.78) and pain (45.5% vs. 54.5%; *p *= 0.62).

### Seasonal distribution of bacterial etiology of episodes

**Figure 3:**
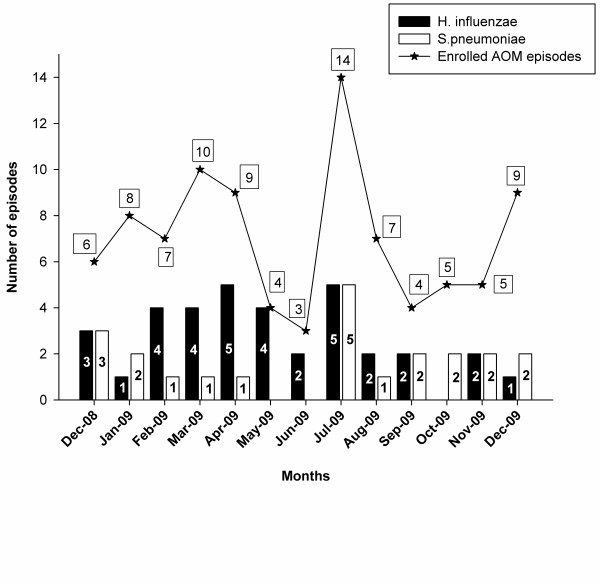
**Seasonal distribution of AOM episodes (ATP Cohort)**. The blue stacked line graph shows the number of AOM episodes enrolled in each month and the simple bar graph shows the number of *H. influenzae *and *S. pneumoniae *episodes isolated each month.

AOM episodes occurred throughout the year; the highest numbers of episodes were recorded for children enrolled in 2009 (July: 14 episodes; March: 10 episodes). The highest number of *H. influenzae *positive episodes was in children enrolled in April and July (14.3% each; 5/35 episodes). At least one *S. pneumoniae *positive episode was reported for most months of the year - between January and April, and July and December, inclusive, the highest number of episodes being enrolled in July (22.7%; 5/22 episodes) (Figure 3).

### Vaccination status

About 80.2% (73/91 episodes) of episodes occurred in children who received at least one dose of the PCV-7. About 97.1% (34/35) subjects testing positive for *H. influenzae *received at least one *H. influenzae type b *vaccine dose.

The percentage of episodes with positive cultures for at least one of the four study bacteriawas 69.6% (32/46 episodes) in fully vaccinated children, 63% (17/27 episodes) in partially vaccinated and 57.1% (8/14 episodes) in unvaccinated children.

More specifically for *S. pneumoniae*, the percentage of culture positive episodes was 30.3% (10/33 episodes), 35.3% (6/17 episodes) and 40.0% (4/10 episodes) in the fully vaccinated, partially vaccinated and unvaccinated children, respectively. The percentage of *H. influenzae *culture positive episodes were 63.6% (21/33 episodes) in the fully vaccinated children, 52.9% (9/17 episodes) in partially vaccinated and 40.0% (4/10 episodes) in unvaccinated children, respectively. We did not find significant differences between capsulated and non-capsulated *H. influenzae *or between fully vaccinated and partially/unvaccinated groups (*p *= 0.38).

### Antibacterial susceptibility

Among the 35 *H. influenzae *positive samples, 94.3% (33/35) were sensitive to amoxicillin/ampicillin, 5.7% (2/35) were resistant to amoxicillin/ampicillin and 2.9% (1/35) showed intermediate resistance to cefuroxime. 25.7% (9/35) samples showed resistance to trimethoprim/sulfamethoxazole. The two isolates resistant to amoxicillin/ampicillin and susceptible to amoxicillin/clavulanate were tested for β-lactamase activity with positive results (n = 2).

**Figure 4:**
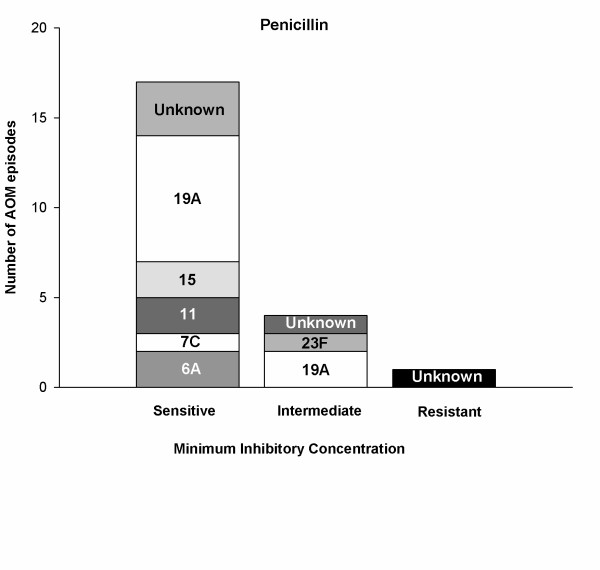
**Penicillin resistance for *S. pneumoniae *isolates (ATP Cohort)**. Susceptibility of each S. pneumoniae serotype to penicillin for categories viz; sensitive, intermediate and resistant.

Among the 22 *S. pneumoniae *positive samples, 77.3% (17/22) were sensitive, 18.2% (4/22) and 4.5% (1/22) of samples were intermediate and resistant to penicillin, respectively (Figure 4). All *S. pneumoniae *isolates tested except three were sensitive to amoxicillin and ampicillin (86.4%; 19/22). Erythromycin resistance was reported in 33.3% (7/22) samples; 35% (7/22) of samples were resistant to tetracycline and 45.5% (10/22) to trimethoprim/sulfamethoxazole.

Of the nine samples positive for *S. pneumoniae *serotype 19A, 77.8% (7/9) were sensitive to penicillin and intermediate resistance was observed for 22.2% (2/9) samples. No resistance to penicillin was noted for this serotype. No antibiotic resistance was observed to cefotaxime, chloramphenicol or levofloxacin.

## Discussion

This is the first study to investigate the bacterial etiology of AOM in a private setting in Venezuela. Our findings are novel in that we identified non-capsulated and capsulated *H. influenzae *as the main pathogens causing AOM in Venezuela. Among the episodes reported,*H. influenzae *(55.5%) and *S. pneumoniae *(34.9%) were the most commonly isolated bacteria. This is in-line with other studies who have reported these bacteria to be frequently isolated in other Latin American countries including Argentina [36], Chile [13], Costa Rica [12,37], Mexico [38] and Colombia [39]. Following the introduction of PCV-7 and the inclusion of the vaccine into the universal immunization program in the United States, a significant decrease in the proportion of *S. pneumoniae *and an increase in *H. influenzae *proportion among MEF isolates have been reported (2003-2006) [40]. A similar pattern was observed in the present study and has been observed in previous studies [10,41] following introduction of the PCV. An increase in the occurrence of non-PCV-7 *S. pneumoniae *serotypes has also been described previously [42].

In the current analysis, we show that *H. influenzae *isolated in Venezuelan children was most commonly NTHi (62.9%). NTHi has previously been reported to be associated with a history of recurrent AOM episodes, treatment failure and AOM within two weeks of completing a course of any antibiotic [43]. The high prevalence of NTHi in this private setting could be explained by the fact that vaccine coverage for PCV was considerably high (63.6%). In this previous study, the authors showed that NTHi was not only the most prevalent cause of recurrent episodes of AOM but also of new cases and was detected across all age groups [44]. This finding differs from earlier reports of NTHi in children 2 years or older [11]. In our present study, most of the episodes we reported were in children younger than 24 months; NTHi was the most prevalent type among all age groups, including 3-11 month olds, who showed an equal distribution with *S. pneumoniae*.

We also report the isolation of capsulated *H. influenzae *including types *d, c, a *and *f*, which has not been reported previously. These were not only isolated in all age groups, but also reported in recurrent episodes. In a recent systematic review for Latin America, capsulated types were not confirmed in MEF samples from AOM etiology studies except for *b *type. This was also consistent with the results from other studies in Europe, such as France, where *H. influenzae *isolates were identified as NTHi. This could be explained by the fact that nasopharyngeal colonization of encapsulated *H influenzae *is uncommon, occurring only in 2-5% of children [45,46]

We found serotype 19A to be the most commonly isolated *S. pneumoniae *serotype (40.9%) from children who were fully vaccinated with the PCV, suggesting that PCV-7 vaccination does not offer a high level of cross-protection against 19A. Recently, in the US and other countries the non-vaccine serotype 19A has been associated with an increase in the proportions of IPD and AOM [10,26,28,41,47]. Other less commonly found bacterial pathogens in this present study were *S. pyogenes *(2.2%) and *M. catarrhalis *(1.1%), similar to other studies [36,48].

We observed a seasonal pattern of AOM in the present study that was similar to that observed in a recent study in Colombia [39]. We found that AOM episodes were reported throughout the year, with the highest number of episodes reported in July, corresponding to the rainy season in Venezuela.

An increased use of antibiotics has been observed in Latin American countries, predominantly in Venezuela [21]. Antibiotic resistance to *S. pneumoniae *and *H. influenzae *has become a serious public health issue, more so in Latin America due to increased *S. pneumoniae *resistance to penicillin and other antibiotics [7]. Seven of nine *S. pneumoniae *19A isolates were sensitive to penicillin in our study, highlighting the low prevalence of penicillin-resistant serotype 19A. In the *H. influenzae *isolates reported, 25.7% were resistant to trimethoprim-sulfamethoxazole and 5.7% to amoxicillin/ampicillin. We did not observe antibiotic resistance to amoxicillin/clavulanate, cefotaxime, chloramphenicol and tetracycline. A limited occurrence of amoxicillin resistance has been previously detected in another multinational study [49]. Hence, amoxicillin and amoxicillin/clavulanate continue to be considered as appropriate treatment options for AOM in children [50]. Reducing antibiotic use by maximizing vaccination uptake may be a useful strategy to eradicate antibiotic-resistant serotypes.

PCVs could be an important tool in reducing the AOM burden. Besides offering protection against the vaccine serotypes, previous studies suggest that PCV7-CRM provides cross-protection against the non-vaccine serotype 6A for IPD and AOM [28,51] and that PCVs with different conjugation methods or carrier proteins may provide modest (though non-significant) protection against 19A disease [52]. Antibiotic resistance and selection pressures are also likely to have an impact on the efficacy of PCVs. Vaccines that may protect against non-capsulated and capsulated *H. influenzae *serotypes may also play an important role to reduce the number of AOM episodes. Efficacies of 35.6% and 35.3% have previously been shown [31] against all *H. influenzae *and NTHi otitis media episodes with a prototype pneumococcal vaccine conjugated to the *H influenzae *protein D. This protein is present in capsulated and non-capsulated isolates and hence may potentially protect against AOM episodes caused by these agents. The proportions of pathogens causing AOM and potentially targeted by each vaccine formulation would be 55% for PHiD-CV and 19% for PCV-13. However, it is important to note that no efficacy or effectiveness data exist for either formulation. Additionally, previous studies with the precursor vaccines (PCV-7; 11-valent pneumococcal protein D conjugate PHiD-CV predecessor vaccine) have demonstrated efficacy of < 100% against the targeted pathogens [28,31]. The precise magnitude of protection is therefore unknown.

This study is limited by a small sample size which affects the precision of the estimates. In addition, the enrolled children were recruited from a private center and thus may not truly represent the Venezuelan population. This is particularly important given the potential differences in exposure and risk factors for otitis media across the country.

## Conclusions

In summary, this was the first active surveillance for AOM etiology in Venezuela in a private setting in a highly PCV vaccinated population. NTHi was the most prevalent etiological agent, suggesting that protection against NTHi is crucial for AOM prevention. Reduced antibiotic use and vaccine with efficacy against *H. influenzae *(beyond type b) and *S. pneumoniae *could be effective strategies for preventing AOM disease among children in Venezuela.

## Abbreviations

AOM: acute otitis media; ATP: according-to-protocol; CI: confidence interval; CLSI: Clinical and Laboratory Standards Institute; CSOM: chronic suppurative otitis media; ENT: Ear: nose: throat; GSK: GlaxoSmithKline; *H. influenzae: Haemophilus influenzae; *IPD: invasive pneumococcal disease; *M. catarrhalis: Moraxella catarrhalis; *MEF: middle ear fluid; NTHi: Non-typeable *Haemophilus influenzae*; PCR: polymerase chain reaction; PCV: pneumococcal conjugate vaccine; PHiD-CV: pneumococcal non-typeable *Haemophilus influenzae *protein-D conjugate vaccine; *S. aureus: Staphylococcus aureus; S. pneumoniae: Streptococcus pneumoniae; S. pyogenes: Streptococcus pyogenes*; SAS: Statistical Analysis System

## Competing interests

LN and JAS received honoraria/paid expert testimony/travel grants from the commercial entity that sponsored the study.

MMC, RDA, EO-B and REC are employees of GlaxoSmithKline Biologicals.

EOB and REC have stock ownership; MMC, and RDA have stock options.

FS, AC, ES, NR, OA, FB and NM: No conflict.

## Authors' contributions

LN and JAS were involved in all processes related to enrolling patients, contributed to study design, interpretation of data, review and comments on all drafts of this paper and gave final approval to submit for publication. FS was the ENT who performed all tympanoscentesis, interpretation of data, review and comments on all drafts of this paper and gave final approval to submit for publication. AC, ES, NR, OA, FB and NM, worked actively in isolation and serotyping all samples, interpretation of data, review and comments on all drafts of this paper and gave final approval to submit for publication. RDA, EOB, REC and MMC contributed to study design, interpretation of data, review and comments on all drafts of this paper and gave final approval to submit for publication.

## Pre-publication history

The pre-publication history for this paper can be accessed here:

http://www.biomedcentral.com/1471-2334/12/40/prepub

## Supplementary Material

Additional file 1 **Table S1**. Demographic characteristics with bacterial etiology of episodes by age and gender (ATP Cohort). Click here for file
