# Supplementary material for: Non-capsulated and capsulated Haemophilus influenzae in children with acute otitis media in Venezuela: a prospective epidemiological study
Source: BMC Infect Dis. 2012 Feb 15;12:40. doi: 10.1186/1471-2334-12-40 (PMC3305597; doi:10.1186/1471-2334-12-40)
Supplement: Additional file 1 — Table S1. Demographic characteristics with bacterial etiology of episodes by age and gender (ATP Cohort). [file 1471-2334-12-40-S1.DOC]

**Table 1 Demographic characteristics with bacterial etiology of episodes by age and gender** (ATP Cohort).

| **AOM Episodes Characteristics** | **Categories** | **Value or n^a^** | **%^b^** |
| --- | --- | --- | --- |
| **Age (months)** |  |  |  |
| Mean |  | 28.3 | - |
| SD |  | 14.78 | - |
| Median |  | 26 | - |
| Min-Max |  | 5–59 | - |
| **Gender** |  |  |  |
| Female^c^ (N = 47) |  |  | 51.6 |
|  | *H. influenzae* | 17 | - |
|  | *S. pneumoniae* | 11 | - |
|  | *S. pyogenes* | 1 | - |
|  | *M. catarrhalis* | 0 | - |
|  | Others/ Negative | 19 | - |
| Male (N = 44) |  |  | 48.4 |
|  | *H. influenzae* | 18 | - |
|  | *S. pneumoniae* | 11 | - |
|  | *S. pyogenes* | 1 | - |
|  | *M. catarrhalis* | 1 | - |
|  | Others/Negative | 13 | - |

N = 91; number of episodes

^a^n = number of episodes in a given category

^b^% = n/Number of samples with results available * 100

^c^It includes 1 episode with more than 1 bacterial isolate (*H. influenzae* + *E. cloacae*)
